# Supplementary material for: TEM8 marks neovasculogenic tumor-initiating cells in triple-negative breast cancer
Source: Nat Commun. 2021 Jul 20;12:4413. doi: 10.1038/s41467-021-24703-7 (PMC8292527; doi:10.1038/s41467-021-24703-7)
Supplement: Supplementary file 1 — Supplementary Information [file 41467_2021_24703_MOESM1_ESM.pdf]

TEM8 marks neovasculogenic tumor-initiating cells in triple-negative breast cancer

Jiahui Xu[1], Xiaoli Yang[1], Qiaodan Deng[1], Cong Yang[2], Dong Wang[3], Guojuan Jiang[1], Xiaohong Yao[4], Xueyan He[1], Jiajun Ding[1], Jiankun Qiang[1], Juchuanli Tu[1], Rui zhang[1], Qun-Ying Lei[1], Zhi-min Shao[1], Xiuwu Bian[4]*, Ronggui Hu[5]*, Lixing Zhang[1]*, Suling Liu[1,6]*

[1] Fudan University Shanghai Cancer Center & Institutes of Biomedical Sciences; Cancer Institutes; Key Laboratory of Breast Cancer in Shanghai; The Shanghai Key Laboratory of Medical Epigenetics; The International Co-laboratory of Medical Epigenetics and Metabolism, Ministry of Science and Technology; Shanghai Medical College; Fudan University, Shanghai 200032, China. [2] School of Medicine, Guizhou University, Guiyang, Guizhou 550002, China. [3] WPI Nano Life Science Institute, Kanazawa University, Kakuma-machi, Kanazawa 920-1192, Japan. [4] Institute of Pathology and Southwest Cancer Center, Southwest Hospital, Third Military Medical University (Army Medical University); Key Laboratory of Tumor Immunopathology, Ministry of Education of China, Chongqing 400038, China. [5] State Key Laboratory of Molecular Biology; CAS Center for Excellence in Molecular Cell Science; Shanghai Institute of Biochemistry and Cell Biology, Chinese Academy of Sciences, Shanghai, 200031 China. *email: bianxiuwu@263.net; coryhu00@gmail.com; zhang_lx@fudan.edu.cn; suling@fudan.edu.cn.

Supplementary Figure and Figure Legends:

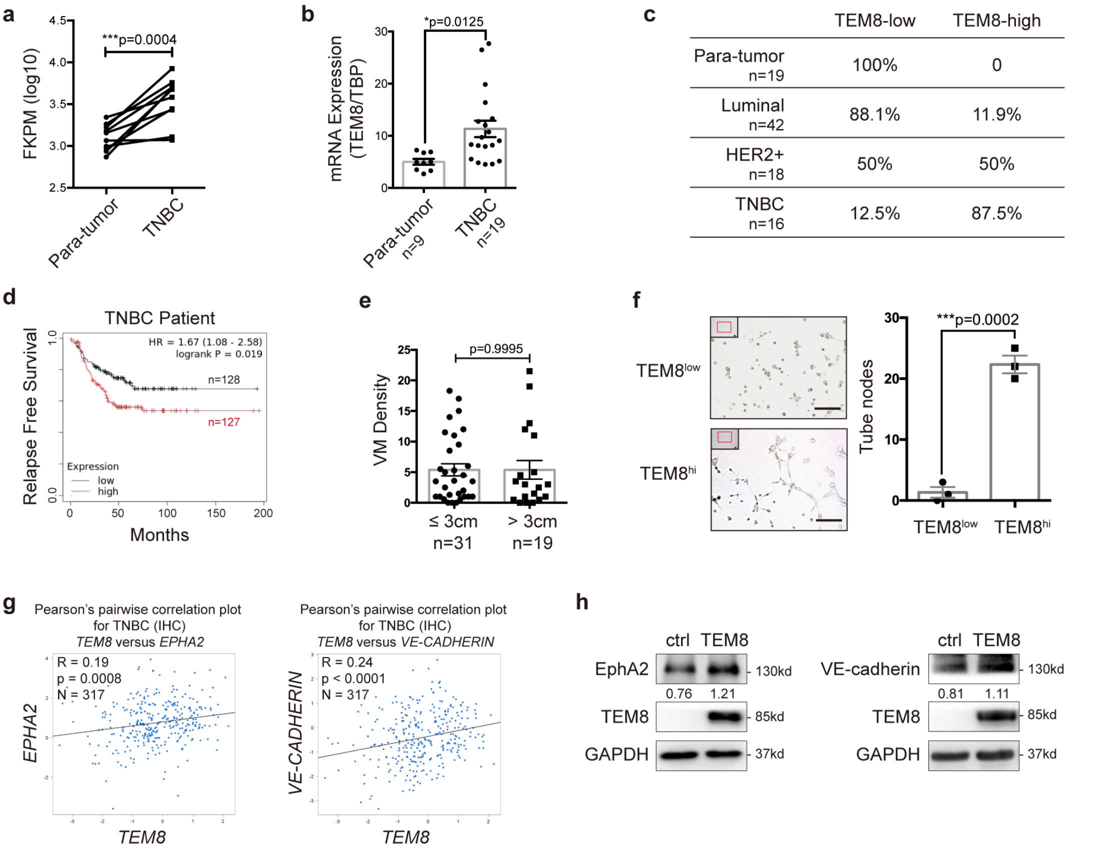

Supplementary Figure 1. TEM8 was highly expressed in TNBC and positively correlated with vascular mimicry.

(**a**) Expression of TEM8 in TNBC tumor and paired para-tumor tissues using the RNA-seq data. (**b**) Expression of TEM8 in TNBC tumor and para-tumor tissues via qRT-PCR. Data were presented as mean ± SEM. (**c**) Percentage of TEM8 low-expressing (H-Score < 150) and high-expressing (H-Score > 150) tissues in different subtypes of BC tissues and para-tumor tissues according to IHC staining in Fig. 1b. (**d**) Analysis of relapse free survival in TNBC patients based on the expression of TEM8. Cohorts were divided at median of TEM8 expression. (**e**) The correlation between the tumor size and the tumor VM density in BC patient tumor samples according to the tumor-associated vessel staining in Fig. 1c. Data were presented as mean ± SEM. (**f**) *In vitro* tube formation analysis of $TEM8^{hi}$ and $TEM8^{low}$ MDA-MB-231^{lung} cells. The graph represented a mean ± SEM of 3 independent experiments. Scale bar, 100μm. (**g**) Correlation analysis of TEM8 and known VM markers (EPHA2 and VE-CADHERIN) using bc-GenExMiner v4.5. (**h**) Western blotting analysis of the expression of EphA2 and VE-cadherin in MDA-MB-231-TEM8 cells. Source data are provided as a Source Data file.

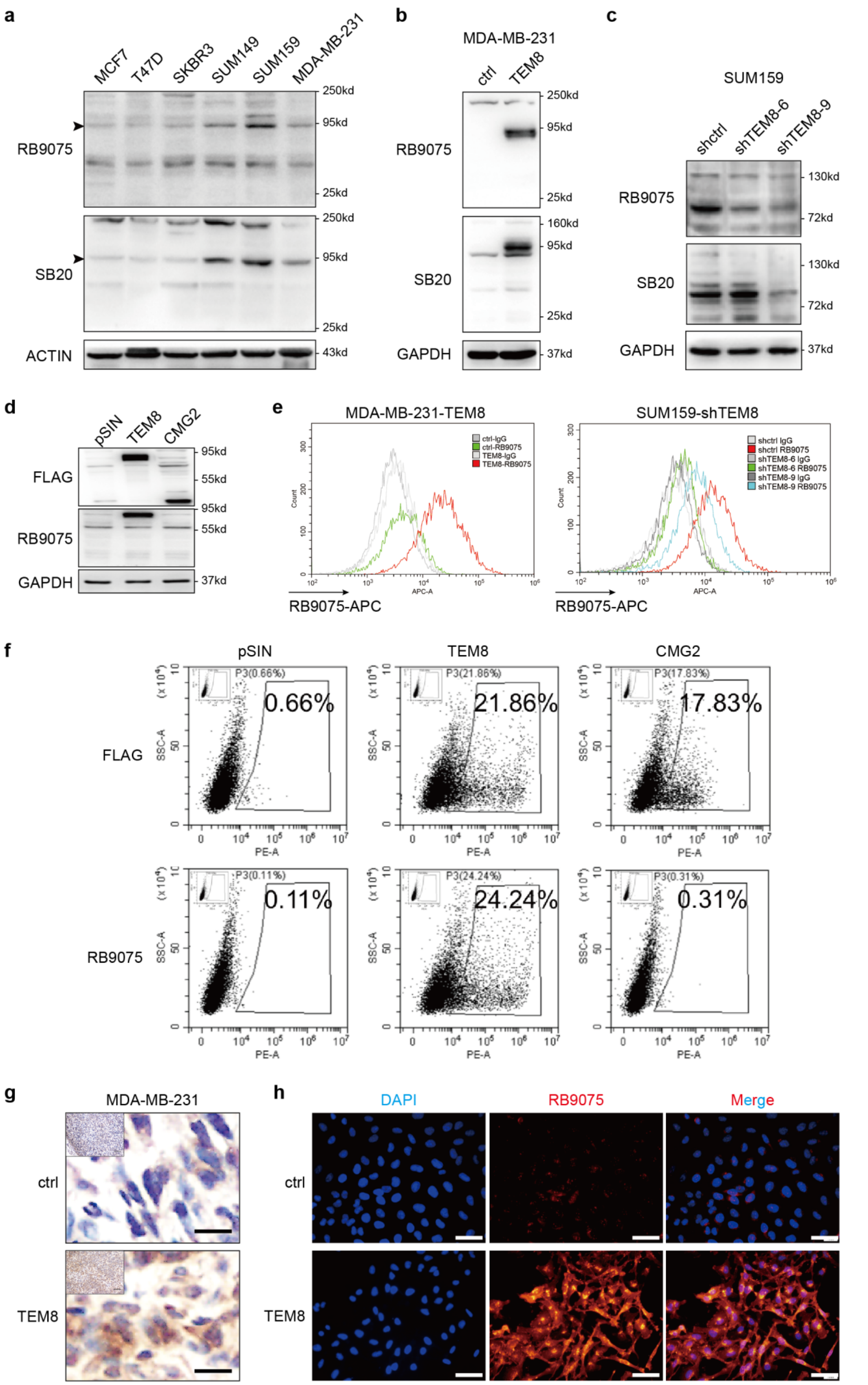

Supplementary Figure 2. The customized TEM8 antibody RB9075 specifically

recognized TEM8.

(**a**) RB9075 antibody was used for western blotting to detect TEM8 expression in BC cell lines. Another commercial TEM8 antibody SB20 was used for the comparison. (**b, c**) RB9075 was used for western blotting to detect the overexpression of TEM8 in MDA-MB-231 cells (b)or knockdown of TEM8 in SUM159 cells (c). (**d**) CMG2 shared a highly similar sequence with TEM8. RB9075 was used for western blotting to detect the overexpression of FLAG-tagged TEM8 and FLAG-tagged CMG2 in 293T cells. (**e**) RB9075 was used for flow cytometry analysis of TEM8 expression in MDA-MB-231-TEM8 or SUM159-shTEM8 cells. IgG was used as a negative control. (**f**) RB9075 was used for flow cytometry analysis of FLAG-tagged TEM8 and FLAG-tagged CMG2 in 293T cells. IgG was used as a negative control. (**g**) RB9075 was used for IHC staining in MDA-MB-231-ctrl/TEM8 cell-derived tumor sections. Scale bar, 20μm. (**h**) RB9075 was used for immunofluorescence staining in MDA-MB-231-TEM8 cells. Scale bar, 100μm. Source data are provided as a Source Data file.

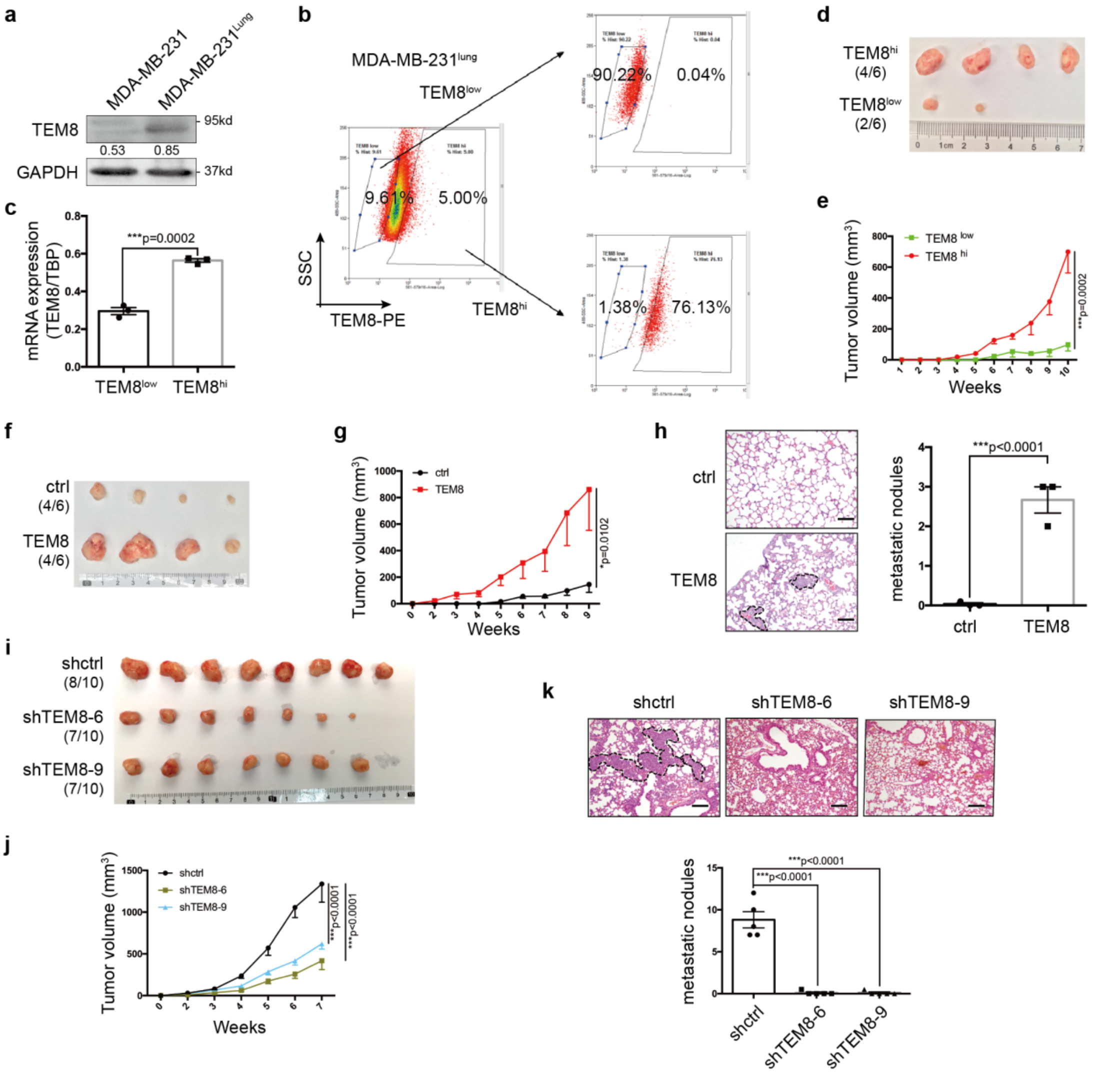

Supplementary Figure 3. TEM8 promoted TNBC cell proliferation and tumor growth.

(**a**) Western blotting analysis of the expression of TEM8 in MDA-MB-231 cells and MDA-MB-231^{lung} cells. (**b**) FACS strategy for TEM8-high and TEM8-low expression MDA-MB-231^{lung} cells. The purity of sorted cells was confirmed by a subsequent flow cytometry detection. (**c**) qRT-PCR analysis of the expression of TEM8 in FACS sorted TEM8^{low} and TEM8^{hi} MDA-MB-231^{lung} cells. The graph represented a mean ± SEM of 3 independent experiments. (**d, e**) FACS sorted TEM8^{low} and TEM8^{hi} MDA-MB-231^{lung} cells were engrafted into mammary fat pads of Nude mice (three animals for each group, 10^4 cells/site). Images of harvested tumors (d) and tumor growth curves (e) were shown. (**f-h**) MDA-MB-231-ctrl/TEM8 cells were engrafted into the mammary fat pads of Nude mice (three animals per group, 10^6 cells/site). Representative image of tumors (f) and the tumor growth curve (g) were shown. HE staining of lung sections and the number of metastatic nodules per lung section were counted (mean ± SEM). Black dotted lines indicated metastases (h). Scale bar, 100μm. (**i-k**) MDA-MB-231^{lung}-shctrl/shTEM8 cells were engrafted into the mammary fat pads of Nude mice (five

animals per group, 10^6 cells/site). Representative image of tumors (i) and the tumor growth curve (j) were shown. HE staining of lung sections and the number of metastatic nodules per lung section were counted (mean ± SEM). Black dotted lines indicated metastases (k). Scale bar, 100μm. Source data are provided as a Source Data file.

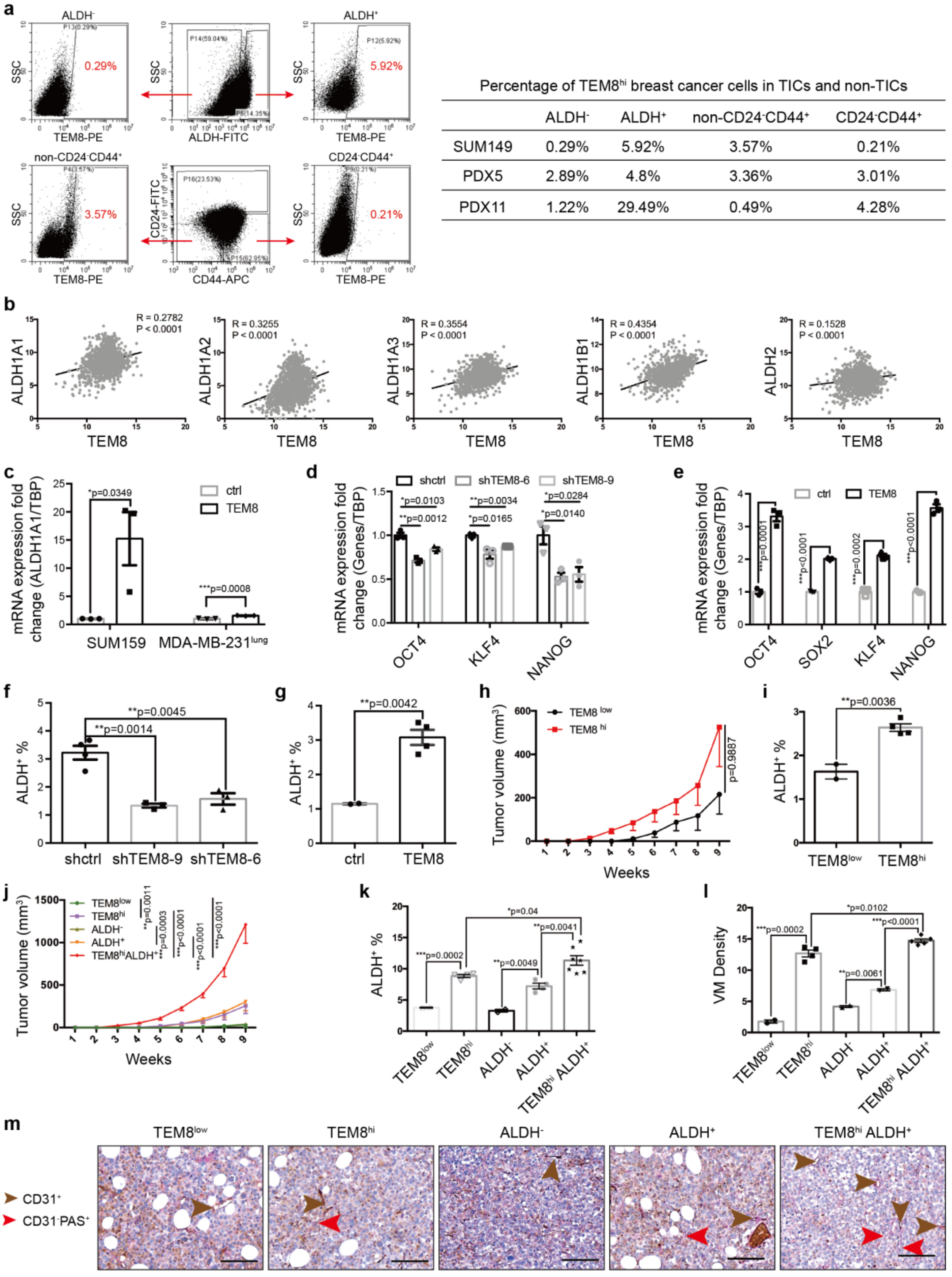

Percentage of TEM8hi breast cancer cells in TICs and non-TICs

	ALDH-	ALDH+	non-CD24-CD44+	CD24-CD44+
SUM149	0.29%	5.92%	3.57%	0.21%
PDX5	2.89%	4.8%	3.36%	3.01%
PDX11	1.22%	29.49%	0.49%	4.28%

n

Confidence interval for 1/(tumor-initiating cell frequency)

Group	Cell Number	Tested	Response	Lower	Estimate	Upper
TEM8low	5000	6	1	215021	30432	4307
	500	6	0			
TEM8hi	5000	6	4	8262	3348	1357
	500	6	2			
ALDH-	5000	6	1	62972	15056	3600
	500	6	1			
ALDH+	5000	6	4	6746	2767	1135
	500	6	3			
TEM8hiALDH+	5000	6	6	758	280	103
	500	6	5			

Pairwise tests for differences in tumor-initiating cell frequencies

Group1	Group2	P-value
TEM8hiALDH+	TEM8low	6.35e-08
TEM8hiALDH+	TEM8hi	0.000426
TEM8hiALDH+	ALDH-	4.3e-07
TEM8hiALDH+	ALDH+	0.000939
ALDH+	TEM8low	0.00513
ALDH+	TEM8hi	0.748
ALDH+	ALDH-	0.0212
TEM8hi	TEM8low	0.0134
TEM8hi	ALDH-	0.0492
ALDH-	TEM8low	0.555

Supplementary Figure 4. TEM8-expressing breast cancer cells exhibited enhanced

tumor-initiating and vasculogenic mimicry forming capacity.

(**a**) Flow cytometry analysis of TEM8 expression in TICs and non-TICs of TNBC cell line SUM149 and TNBC PDXs. Representative images of SUM149 were shown in the left panel. Statistical results were listed in the right panel. (**b**) Correlation analysis of the expression of TEM8 and ALDH isoforms in TCGA database. (**c**) qRT-PCR analysis of the expression of ALDH1A1 in TEM8-overexpressing BC cells. The graph represented a mean ± SEM of 3 independent experiments. (**d, e**) qRT-PCR analysis of the expression of stem cell factors in MDA-MB-231^{lung}-shTEM8 cells (d) and MDA-MB-231-TEM8 cells (e). The graph represented a mean ± SEM of 3 independent experiments. (**f, g**) ALDH activity analyses in MDA-MB-231^{lung}-shTEM8 (f) or MDA-MB-231-TEM8 (g) cell-derived xenografts (five nude mice each group for MDA-MB-231^{lung}-shTEM8, three nude mice each group for MDA-MB-231-TEM8). Bar plots (mean ± SEM) of the percentages of ALDH^{+} tumor cells were shown. (**h, i**) TEM8^{hi} and TEM8^{low} cells sorted from TNBC PDX11 were injected into mammary gland fat pads of NOD/SCID mice (related to Fig. 2j). Tumor growth curve (10^5 cells/site) was shown (h). The bar plot (mean ± SEM) of percentages of ALDH^{+} tumor cells was shown (i). (**j-n**) Cell groups as indicated sorted from MDA-MB-231^{lung} cells were engrafted in limited dilution to mammary fat pads of NOD/SCID mice. The tumor growth curve (5000 cells/site) was shown (j). The percentages of ALDH^{+} tumor cells were analyzed by ALDEFLUOR assay and the bar plot (mean ± SEM) was shown (k). The tumor vasculogenic mimicry was immunostained and quantified. The bar plot (mean ± SEM) of VM density (l) and the representative images of the tumor vasculogenic mimicry (m) were shown. Scale bar, 100μm. The stem cell frequencies were calculated based on the positive tumor sites (n). Source data are provided as a Source Data file.

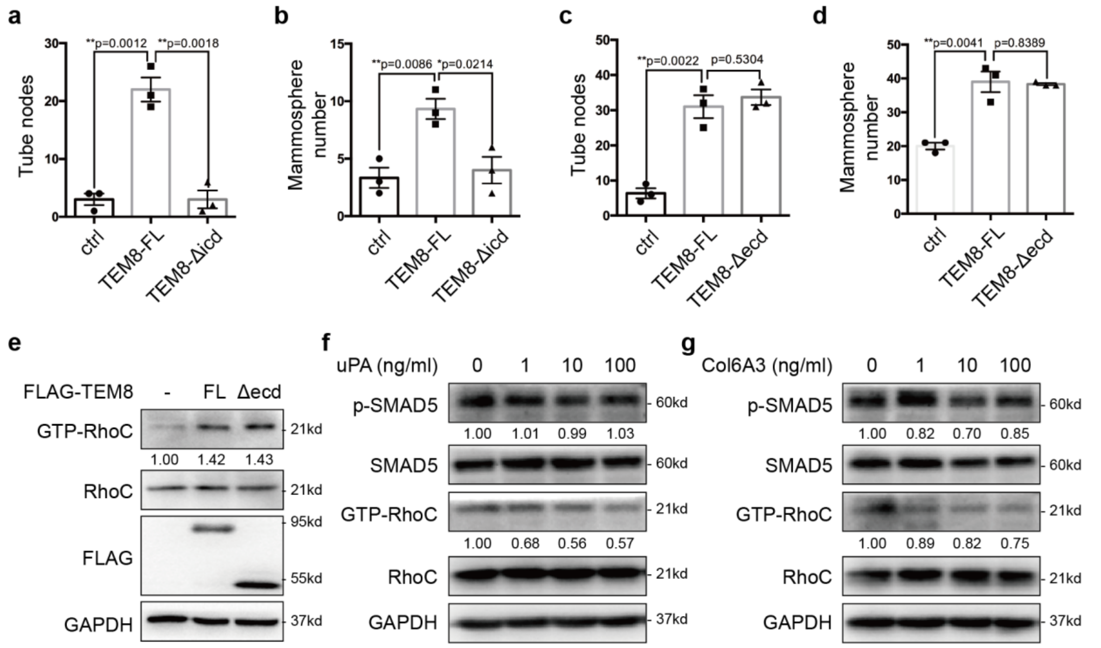

Supplementary Figure 5. The malignant functions of TEM8 was intracellular domain-dependent.

(**a, b**) Full length of TEM8 protein (TEM8-FL) and truncation with intracellular domain deleting (TEM8-Δicd) were stably transfected into MDA-MB-231 cells. The tumor cell VM formation (a) and mammosphere formation (b) were then detected. The graph represented a mean ± SEM of 3 independent experiments. (**c-e**) Full length of TEM8 protein and truncation with extracellular domain deleting (TEM8-Δecd) were stably transfected into MDA-MB-231 cells. The tumor cell VM formation (c), mammosphere formation (d) and active RhoC (e) were then detected and quantitated. The graph represented a mean ± SEM of 3 independent experiments. (**f, g**) Western blotting analysis of RhoC and SMAD5 protein activation in MDA-MB-231-TEM8 cells after uPA (f) and Col6A3 (g) stimulation for 1 hour. Source data are provided as a Source Data file.

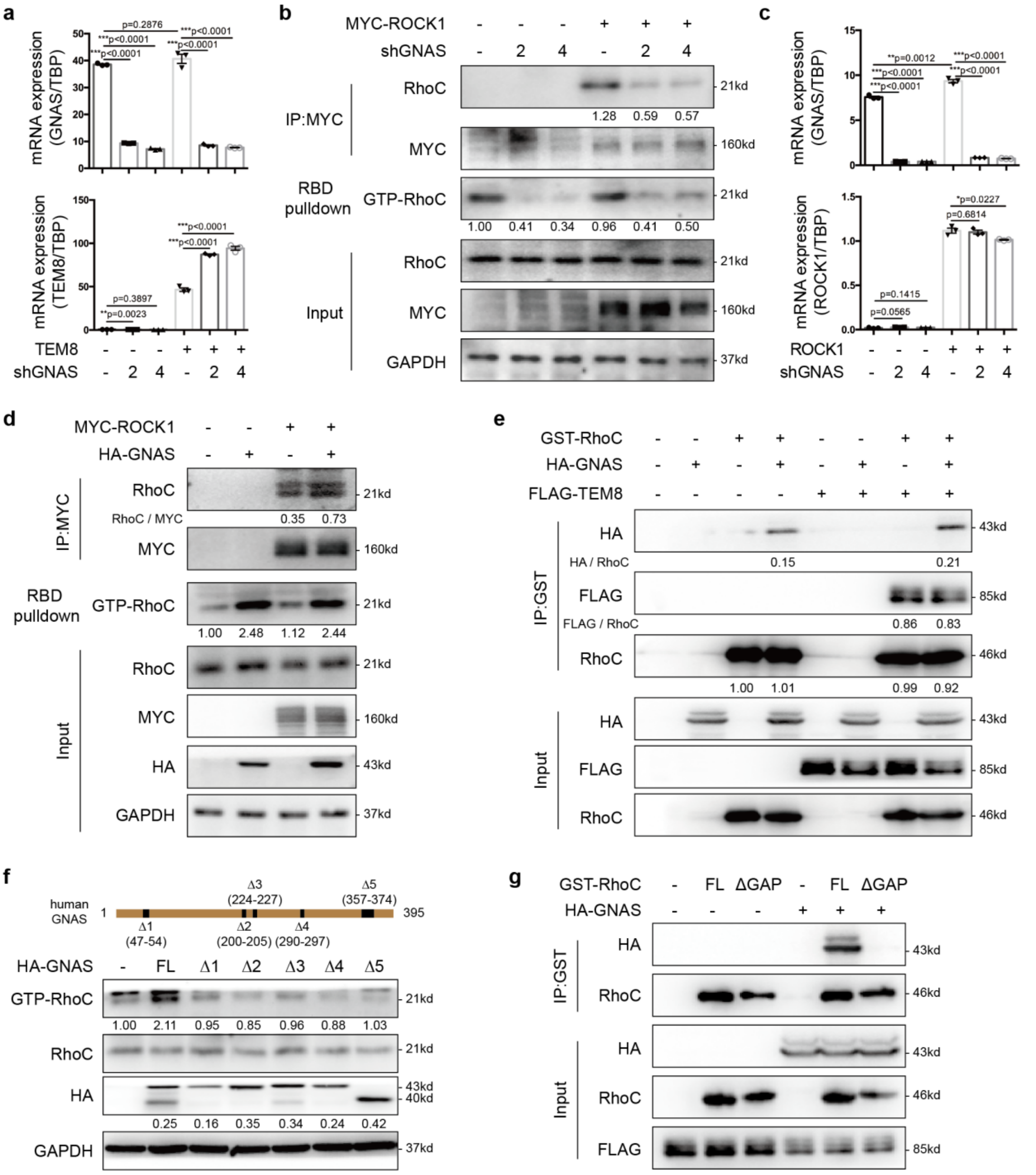

Supplementary Figure 6. GNAS bound to RhoC GAP binding site via its GTP binding site.

(a) Efficiency of GNAS knockdown and TEM8 overexpression in 293T cells was quantitated by RT-qPCR. The graph represented a mean ± SEM of 3 independent experiments. (b) GNAS/scrambled shRNA and ROCK1 were transfected into 293T cells as indicated. The interaction between ROCK1 and RhoC and the active RhoC level were then determined and quantitated. (c) Efficiency of GNAS knockdown and ROCK1 overexpression in 293T cells was quantitated by RT-qPCR. The graph represented a mean ± SEM of 3 independent experiments. (d) GNAS and ROCK1 were transfected into 293T cells as indicated. The interaction between ROCK1 and RhoC and the active RhoC level were then determined and quantitated. (e) The interaction between RhoC and GNAS in the presence or absence of TEM8 protein was analyzed by in vitro co-IP assay. FLAG and HA IP bands were quantitated to the corresponding RhoC IP bands.

(f) Full length GNAS protein (FL) and GTP-binding site-deleted truncates were transfected into 293T cells. The active RhoC was then determined and quantitated to total RhoC. (g) Full length RhoC (FL) and GAP binding site-deleted truncate (ΔGAP) proteins were purified. The interaction between GNAS and RhoC was analyzed by in vitro co-IP assay. Source data are provided as a Source Data file.

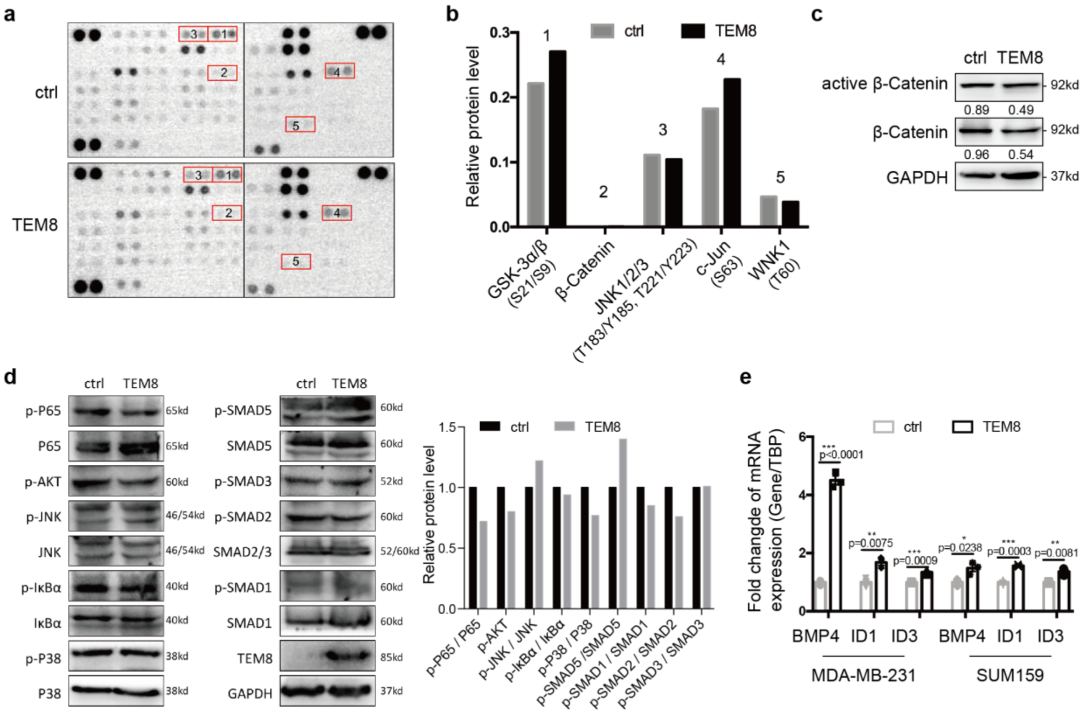

Supplementary Figure 7. SMAD5 pathway was activated in TEM8-overexpressing TNBC cells.

(**a**) Analyzing the phosphorylation profile of kinases in MDA-MB-231-TEM8 cells using a human phospho-kinase array. Representative images of the array were shown. (**b**) Quantification of the expression level of Wnt pathway associated kinases in MDA-MB-231-TEM8 cells detected by the human phospho-kinase array. (**c**) western blotting analyses of the total β-Catenin and active β-Catenin (non-phospho at S33/37/T41) protein level in MDA-MB-231-TEM8 cells. (**d**) western blotting analyses of the ROCK1 associated downstream pathways in MDA-MB-231-TEM8 cells. (**e**) qRT-PCR analyses of SMAD5 downstream target genes in TEM8-overexpressing cells. The graph represented a mean ± SEM of 3 independent experiments. Source data are provided as a Source Data file.

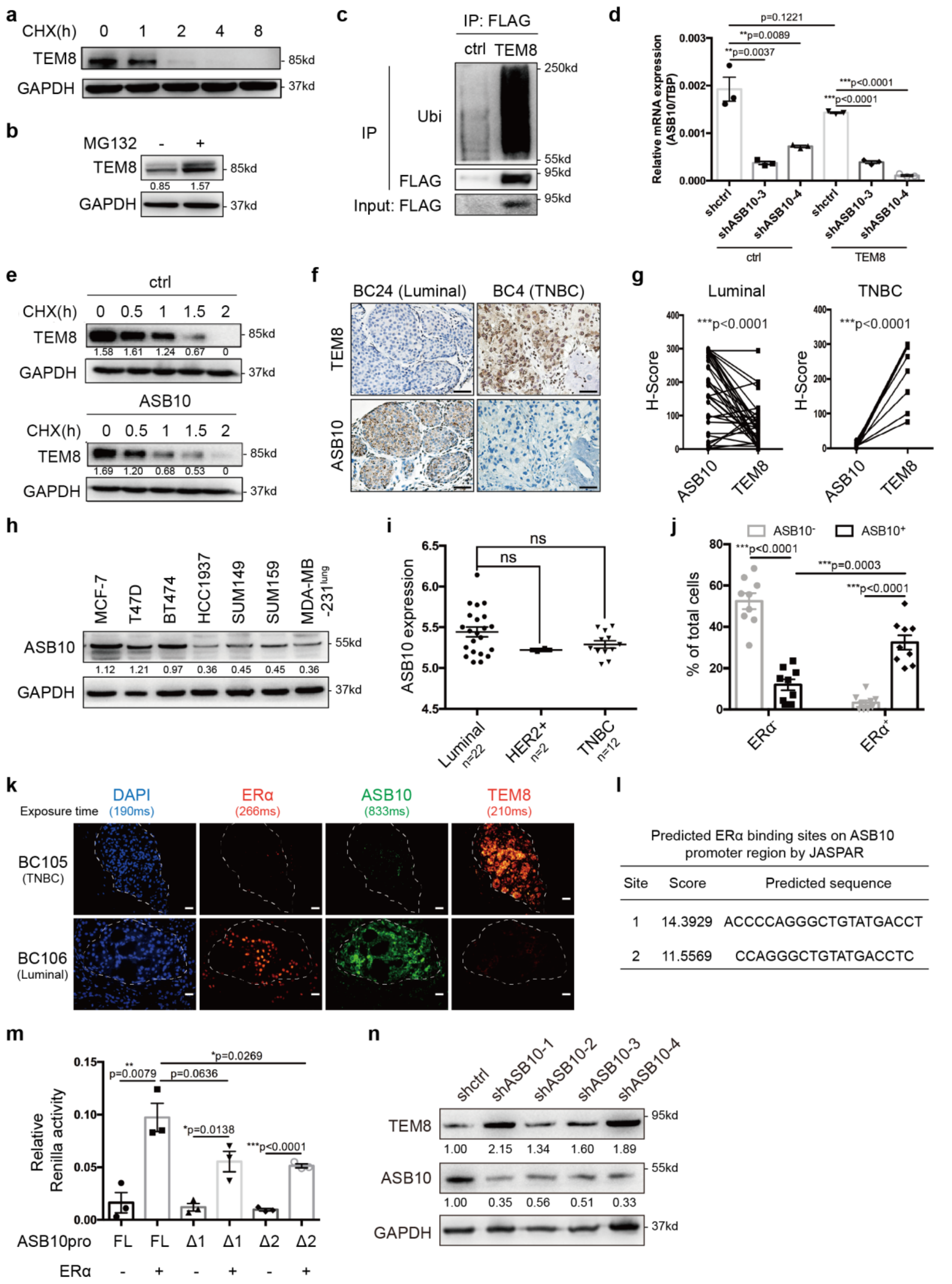

Supplementary Figure 8. ERα-transactivated E3 ligase ASB10 was responsible for TEM8 ubiquitination.

(a) western blotting analysis of the degradation of TEM8 protein in MDA-MB-231-TEM8 cells under CHX treatment. (**b**) western blotting analysis of the TEM8 protein level in MDA-MB-231-TEM8 cells under MG132 treatment. (**c**) Ubiquitination of TEM8 protein (FLAG-tagged) was analyzed by IP and subsequent western blotting. (**d**) Efficiency of ASB10 knockdown in MDA-MB-231-ctrl/TEM8 cells. The graph

represented a mean ± SEM of 3 independent experiments. (**e**) ASB10 was transfected into MDA-MB-231-TEM8 cells and the degradation of TEM8 protein was then analyzed under CHX treatment. (**f**) Representative images of TEM8 and ASB10 expression analyzed by IHC in sequential sections of breast tumors. Scale bar, 50μm. (**g**) Correlation analysis of ASB10 H-Score (related to Fig. 6i) and TEM8 H-Score (related to Fig. 1b) in luminal and TNBC subtypes. (**h**) western blotting analysis of the expression of ASB10 in BC cell lines. (**i**) Analysis of the expression of ASB10 in different subtypes of BC (n=36 biological independent samples) based on TCGA database. Data were presented as mean ± SEM. (**j**) Distribution of ASB10 and ERα in Fig. 6j was shown. The graph represented a mean ± SEM of 9 biological independent samples. (**k**) Fluorescence immunostaining of ERα, ASB10 and TEM8 in sequential sections of breast tumors. Scale bar, 50μm. (**l**) Predicted ERα binding sites on ASB10 promoter region (2000bp upstream of transcription start site) by JASPAR. (**m**) ASB10 promoter truncations with ERα binding sites deletion were constructed and transfected into 293T cells. Dual luciferase reporter assay was then used to evaluate the binding ability of ERα to ASB10 promoter. The graph represented a mean ± SEM of 3 independent experiments. (**n**) Western blotting analysis of TEM8 protein in ASB10 knockdown MCF-7 cells. Source data are provided as a Source Data file.

Supplementary Table 1. Clinicopathological variables and expression of TEM8 in the study cohort

Variables	Number of patients N (%)	TEM8 expression Low N (%)	High N (%)	P[a] value
Total	76	48 (63.2)	28 (36.8)	
Age				0.221
≤50 years	26 (34.2)	19 (25)	7 (9.2)	
>50years	50 (65.8)	29 (34.2)	21 (27.6)	
Tumor size				0.342
≤3cm	48 (63.2)	32 (42.1)	16 (21.1)	
＞3cm	27 (35.5)	16 (21.1)	11 (14.5)	
Unknown	1 (1.3)	0 (0)	1 (1.3)	
Lymph node status				0.773
Negative	30 (39.5)	19 (25)	11 (14.5)	
Positive	39 (51.3)	26 (34.2)	13 (17.1)	
Unknown	7 (9.2)	3 (3.9)	4 (5.3)	
Grade				*0.0316
II	19 (25)	16 (21.1)	3 (3.9)	
III	53 (69.7)	30 (39.4)	23 (30.3)	
Unknown	4 (5.3)	3 (3.9)	1 (1.3)	
ER status				***<0.001
Negative	35 (46.1)	12 (15.8)	23 (30.3)	
Positive	41 (53.9)	37 (48.7)	4 (5.3)	
PR status				***<0.001
Negative	40 (52.6)	16 (21.1)	24 (31.6)	
Positive	36 (47.4)	32 (42.1)	4 (5.3)	
HER2 status				1.000
Negative	50 (65.8)	32 (42.1)	18 (23.7)	
Positive	25 (32.9)	16 (21.1)	9 (11.8)	
Unknown	1 (1.3)	0 (0)	1 (1.3)	
Subtype[b]				***<0.001
Luminal	42 (55.3)	37 (48.7)	5 (6.6)	
HER-2 Enrichment	18 (23.7)	9 (11.8)	9 (11.8)	
TNBC	16 (21.1)	2 (2.6)	14 (18.4)	

Abbreviations: TEM8, tumor endothlial marker 8; ER, estrogen receptor; PR, progesterone receptor; HER-2, human epidermal growth factor receptor 2; TNBC, triple negative breast cancer;

[a] Based on Pearson $\chi 2$ test (Fisher exact test was used when needed).

[b] Definition of subtypes: Luminal (ER and/or PR positive), HER-2 Enrichment (ER and PR negative, HER-2 positive), and TNBC (ER negative, PR negative, and HER-2 negative)

Supplementary Table 2. Potential TEM8-interacting proteins identified by MS

Protein	SUM159 -ctrl	SUM159 -TEM8	MDA-MB-231 -ctrl	MDA-MB-231 -TEM8
ANTXR1	0	15017000000	0	12352000000
TUBB6	0	45307000	0	31474000
ESYT1	0	5818600	0	6079900
DHX38	0	4098400	0	1158300
BZW1	0	3063800	0	4663900
RAB18	0	5905600	0	4376300
RHOC	0	7235900	0	5581700
UTS2	0	8375700	0	10541000
RAB10	0	9473700	0	4133300
S100A10	0	6541400	0	4219700
PTPN1	0	27870000	0	27459000
EEF2	0	10938000	0	3676200
CNP	0	3909000	0	21834000
MT-CO2	0	26364000	0	14863000
ACSL4	0	7093700	0	4723800
PANK4	0	1320100	0	1330800
COX7A2	0	9788800	0	11782000
GNAS	0	4056600	0	2916900
TPM3	0	3016300	0	1425500
ATPIF1	0	2629500	0	285420
BAT3	0	39891000	0	25634000
TJP2	0	1219100	0	1472400

Supplementary Table 3. shRNA sequence information used in this study

Target	Sequence (5' → 3')
shTEM8-6	CCGGCCCACAGTTGAGAATGTCCTTCTCGAGAAGGACATTCTCAACTGTGGGTTTTTG
shTEM8-9	CCGGACACTCAATGAGAAGCCCTTTCTCGAGAAAGGGCTTCTCATTGAGTGTTTTTG
shRhoC-1	CCGGCTACTGTCTTTGAGAACTATACTCGAGTATAGTTCTCAAAGACAGTAGTTTTTG
shRhoC-2	CCGGTGATGTCATCCTCATGTGCTTCTCGAGAAGCACATGAGGATGACATCATTTTTG
shRhoC-3	CCGGGAATAAGAAGGACCTGAGGCACTCGAGTGCCTCAGGTCCTTCTTATTCTTTTTG
shGANS-1	CCGGGCCAAGTACTTCATTCGAGATCTCGAGATCTCGAATGAAGTACTTGGCTTTTTG
shGNAS-2	CCGGCCTCCCGAATTCTATGAGCATCTCGAGATGCTCATAGAATTCGGGAGGTTTTTG
shGNAS-3	CCGGCAGAATTTGCTCGCTACACTACTCGAGTAGTGTAGCGAGCAAATTCTGTTTTTG
shGNAS-4	CCGGCAACGATGTGACTGCCATCATCTCGAGATGATGGCAGTCACATCGTTGTTTTTG
shROCK1-2	CCGGCCCGATTTAAGTAGTGACATTCTCGAGAATGTCACTACTTAAATCGGGTTTTT
shROCK1-5	CCGGGCCAGCAAAGAGAGTGATATTCTCGAGAATATCACTCTCTTTGCTGGCTTTTT
shSMAD5-4	CCGGGCCTAAACATTGGTGTTCAATCTCGAGATTGAACACCAATGTTTAGGCTTTTTG
shSMAD5-5	CCGGCCGTTGGATATTTGTGAATTTCTCGAGAAATTCACAAATATCCAACGGTTTTTG
shASB10-3	CCGGGAGACTCTGGTCTCTGACATACTCGAGTATGTCAGAGACCAGAGTCTCTTTTTG
shASB10-4	CCGGGCATGTCCTGATGCCCGCAATCTCGAGATTGCGGGCATCAGGACATGCTTTTTG

Supplementary Table 4. Primer sequence information for qRT-PCR

Target		Sequence (5' → 3')
TEM8	Forward	GGCTTCCAGTGGCTCTCTTT
	Reverse	ACAGGTCAAATCCGCCGTAG
OCT4	Forward	CTGGGTTGATCCTCGGACCT
	Reverse	CACAGAACTCATACGGCGGG
SOX2	Forward	GCACATGAACGGCTGGAGCAACG
	Reverse	TGCTGCGAGTAGGACATGCTGTAGG
KLF4	Forward	CCCCACCTTCTTCACCCCTAGA
	Reverse	GTAAGGTTTCTCACCTGTGTGGG
NANOG	Forward	AATACCTCAGCCTCCAGCAGATG
	Reverse	TGCGTCACACCATTGCTATTCTTC
RhoC	Forward	GGAGGTCTACGTCCCTACTGT
	Reverse	CGCAGTCGATCATAGTCTTCC
GNAS	Forward	TGCAAGGAGCAACAGCGAT
	Reverse	GCGGCCACAATGGTTTCAAT
FOSB	Forward	GCTGCAAGATCCCCTACGAAG
	Reverse	ACGAAGAAGTGTACGAAGGGTT
BMP4	Forward	TAGCAAGAGTGCCGTCATTCC
	Reverse	GCGCTCAGGATACTCAAGACC
ID1	Forward	CTGCTCTACGACATGAACGG
	Reverse	GAAGGTCCCTGATGTAGTCGAT
ID3	Forward	GAGAGGCACTCAGCTTAGCC
	Reverse	TCCTTTTGTCGTTGGAGATGAC
ROCK1	Forward	AACATGCTGCTGGATAAATCTGG
	Reverse	TGTATCACATCGTACCATGCCT
SMAD5	Forward	TCTCCAAACAGCCCTTATCCC
	Reverse	GCAGGAGGAGGCGTATCAG
ASB10	Forward	TTGGAGCGAGAGTGGATGGT
	Reverse	CTCGGCATCGGTGATGGAC
ASB10pro-primer1	Forward	TTAAGCCAGCGTGAGCCAAAGGG
	Reverse	TGACCCACCACTGCCCACC
ASB10pro-primer2	Forward	ACCCGGCCCCCAAACAAAA
	Reverse	CCCCTGCTGTGGTTACATGGG

Supplementary Table 5. The antibody information used in this study

For Western blotting and IP			
Antibody	Dilution	Catalog	Manufacturer
RB9075	WB 1:500	-	customized
FLAG	WB 1:1000, IP 1:50	F7425	Sigma-Aldrich
HA	WB 1:1000, IP 1:50	3724	CST
ASB10	WB 1:1000	H00136371-M02	Novus
Ubiquitin	WB 1:500	sc-8017	SantaCruz
SMAD5	WB 1:1000	12534	CST
phospho-SMAD1/5	WB 1:1000	9516	CST
SMAD1	WB 1:1000	6944	CST
phospho-SMAD1	WB 1:1000	5753	CST
SMAD2/3	WB 1:1000	8685	CST
phospho-SMAD2	WB 1:1000	8685	CST
phospho-SMAD3	WB 1:1000	9520	CST
GAPDH	WB 1:1000	HC301	Transgen
RhoC	WB 1:1000	3430	CST
VE-Cadherin	WB 1:1000	sc-9989	SantaCruz
P65	WB 1:1000	8242	CST
phospho-P65	WB 1:1000	3033	CST
phospho-Akt	WB 1:1000	4060	CST
JNK	WB 1:1000	9252	CST
phospho-JNK	WB 1:1000	9251	CST
IκBα	WB 1:1000	4812	CST
phospho-IκBα	WB 1:1000	2859	CST
P38	WB 1:1000	9212	CST
phospho-P38	WB 1:1000	4631	CST
β-Catenin	WB 1:1000	8480	CST
Non-phospho β-Catenin	WB 1:1000	8814	CST

HRP-conjugated anti-Rabbit IgG	WB 1:10000	HS101	Transgen
HRP-conjugated anti-Mouse IgG	WB 1:10000	HS201	Transgen

For IHC and immunofluorescence

Antibody	Dilution	Catalog	Manufacturer
RB9075	IHC 1:50, IF 1:50	-	customized
CD31	IHC 1:50, IF 1:50	ab28364	Abcam
	IHC 1:50, IF 1:50	sc-376764	SantaCruz
	IHC 1:50, IF 1:50	sc-18916	SantaCruz
ASB10	IHC 1:100, IF 1:100	H00136371-M02	Novus
ERα	IF 1:200	ab241557	Abcam
		ZA-0102	Zsbio
RhoC	IHC 1:100, IF 1:100	3430	CST
EphA2	IF 1:100	sc-398832	SantaCruz
NG2	IF 1:100	sc-53389	SantaCruz
FLAG	IHC 1:100, IF 1:100	F7425	Sigma-Aldrich
HA	IF 1:100	3724	CST
Alexa Fluor 647 Isolectin IB4	IF 1:200	I32450	Invitrogen
Alexa Fluor 488 anti-Mouse IgG	IF 1:200	A11001	Invitrogen
Alexa Fluor 546 anti-Mouse IgG	IF 1:200	A11003	Invitrogen
Alexa Fluor 488 anti-Rabbit IgG	IF 1:200	A11008	Invitrogen
Alexa Fluor 546 anti-Rabbit IgG	IF 1:200	A11035	Invitrogen
Alexa Fluor 647 anti-Mouse IgG	IF 1:200	715-606-150	Jackson ImmunoResearch
Alexa Fluor 555 anti-Rat IgG	IF 1:200	A21434	Invitrogen

For flow cytometry staining

Antibody	Dilution	Catalog	Manufacturer

H2-Kd	1:200	116607	Biolegend
FLAG	1:50	F7425	Sigma-Aldrich
TEM8	1:50	-	customized
PE-conjugated donkey anti-rabbit IgG	1:200	711-116-152	Jackson ImmunoResearch
APC-conjugated donkey anti-rabbit IgG	1:200	711-136-152	Jackson ImmunoResearch
