# Supplementary material for: TEM8 marks neovasculogenic tumor-initiating cells in triple-negative breast cancer
Source: Nat Commun. 2021 Jul 20;12:4413. doi: 10.1038/s41467-021-24703-7 (PMC8292527; doi:10.1038/s41467-021-24703-7)
Supplement: Supplementary file 3 — Reporting Summary [file 41467_2021_24703_MOESM3_ESM.pdf]

nature research

Corresponding author(s): Suling Liu

Last updated by author(s): Jun 23, 2021

Reporting Summary

Nature Research wishes to improve the reproducibility of the work that we publish. This form provides structure for consistency and transparency in reporting. For further information on Nature Research policies, see our Editorial Policies and the Editorial Policy Checklist.

Statistics

For all statistical analyses, confirm that the following items are present in the figure legend, table legend, main text, or Methods section.

n/a	Confirmed	
☐	☒	The exact sample size (*n*) for each experimental group/condition, given as a discrete number and unit of measurement
☐	☒	A statement on whether measurements were taken from distinct samples or whether the same sample was measured repeatedly
☐	☒	The statistical test(s) used AND whether they are one- or two-sided<br>*Only common tests should be described solely by name; describe more complex techniques in the Methods section.*
☐	☒	A description of all covariates tested
☐	☒	A description of any assumptions or corrections, such as tests of normality and adjustment for multiple comparisons
☐	☒	A full description of the statistical parameters including central tendency (e.g. means) or other basic estimates (e.g. regression coefficient) AND variation (e.g. standard deviation) or associated estimates of uncertainty (e.g. confidence intervals)
☐	☒	For null hypothesis testing, the test statistic (e.g. *F*, *t*, *r*) with confidence intervals, effect sizes, degrees of freedom and *P* value noted<br>*Give P values as exact values whenever suitable.*
☒	☐	For Bayesian analysis, information on the choice of priors and Markov chain Monte Carlo settings
☒	☐	For hierarchical and complex designs, identification of the appropriate level for tests and full reporting of outcomes
☐	☒	Estimates of effect sizes (e.g. Cohen's *d*, Pearson's *r*), indicating how they were calculated

Our web collection on statistics for biologists contains articles on many of the points above.

Software and code

Policy information about availability of computer code

Data collection	MoFlo Astrios instrument (Beckman Coulter, Brea, USA),<br>Applied Biosystems 7300Plus Real Time PCR system(ABI):Collecting qRT-PCR data,<br>Image Quant LAS 4000 mini-imaging system (GE, Fairfield, USA): Collecting the Western Blot data,<br>Mass spectrometry (Orbitrap Elite): Collecting the data of initial protein profiling experiment,<br>Confocal Laser Scanning Microscope (LSM710, Zeiss): Collecting the immunofluorescence data,<br>2100 Bioanalyzer (Agilent, Santa Clara, CA, USA): measuring the RNA concentration and quality,<br>HiSeq3000 platform (Illumina, San Diego, CA, USA): RNA sequencing.
Data analysis	Most of the statistical analyses were performed by GraphPad Prism 6.0 and ImageJ 1.50g (Java 1.8.0_181) software,<br>ALDH and TEM8 analysis and cell sorting were performed using Summit 6.3 software,<br>GSEA (GSEA v3.0) was also used for gene expression difference analysis,<br>Primer design was performed by Primer Bank (https://pga.mgh.harvard.edu/primerbank/) and Primer Blast((https://www.ncbi.nlm.nih.gov/tools/primerblast/index.cgi?LINK_LOC=BlastHome)),<br>HISAT2 version 2.0.0-beta was used to align reads onto hg19 reference genome with default settings,<br>Gene counts were given by featureCounts Version 2.0.0.

For manuscripts utilizing custom algorithms or software that are central to the research but not yet described in published literature, software must be made available to editors and reviewers. We strongly encourage code deposition in a community repository (e.g. GitHub). See the Nature Research guidelines for submitting code & software for further information.

Data

Policy information about availability of data

All manuscripts must include a data availability statement. This statement should provide the following information, where applicable:

- Accession codes, unique identifiers, or web links for publicly available datasets
- A list of figures that have associated raw data
- A description of any restrictions on data availability

The data from TCGA analyzed in the study are available in a public repository from the National Cancer Institute Cancer Genome Atlas website (https://tcga-data.nci.nih.gov/tcga/). The RNA-seq data profiles analyzed in the study have been deposited in the National Center for Biotechnology Information under the accession code PRJNA739366 (https://www.ncbi.nlm.nih.gov/bioproject/PRJNA739366/). The publicly available dataset used in this study can be accessed under the GEO accession codes-GSE43742, GSE86788, GSE5327, GSE2603, GSE2034, GSE22133. All other data supporting the findings of the study are available in this article and its supplementary information files. All other relevant data are available from the corresponding author on request.

Field-specific reporting

Please select the one below that is the best fit for your research. If you are not sure, read the appropriate sections before making your selection.

- [x] Life sciences
- [ ] Behavioural & social sciences
- [ ] Ecological, evolutionary & environmental sciences

For a reference copy of the document with all sections, see nature.com/documents/nr-reporting-summary-flat.pdf

Life sciences study design

All studies must disclose on these points even when the disclosure is negative.

Sample size	For in vitro tumor cell tube formation assay, the number of implanted cells on growth factor reduced matrigel was based on prior experience of HUVEC cell tube formation. For in vitro mammosphere formation assay, the number of implanted cells into low attachment surface plate was depended on the size of plate. For in vitro invasion assay and MTT assay, the number of implanted cells was based on prior experience. For in vitro human kinase array assay and dual luciferase reporter assay, the sample size was determined based on the manufacturer's protocol. For in vivo validation experiments, the number of mice used in each experimental group was determined by power analysis and on the basis of prior experience with animal models of xenografts. Animals were randomly assigned to treatment groups. All sample sizes are listed in the corresponding figure legends or on the figures.
Data exclusions	No data was excluded from the analyses
Replication	All experiments were conducted at least three times independently, and similar results were adopted for further analysis to guarantee reproducibility.
Randomization	For in vitro studies, the samples/cells were randomized into different groups prior to treatment. For animal experiments, all mouse were randomly divided into each group before receiving different treatment.
Blinding	This study included a lot of complicated experimental design, the researchers were limited, and the feasibility of blinding was poor, thus blinding was not efficiently applied. Investigators were blinded to the in vivo xenograft, drug treatment experiments and other in vitro experiments which conducted on cell lines or PDX tumor cells. Investigators were blinded to test and analysis of tumor vasculatures from xenografted tumors by immunostaining. Investigators were not blinded to the Western blot experiments as the researchers need to rank and load the samples based on the treatment information.

Reporting for specific materials, systems and methods

We require information from authors about some types of materials, experimental systems and methods used in many studies. Here, indicate whether each material, system or method listed is relevant to your study. If you are not sure if a list item applies to your research, read the appropriate section before selecting a response.

Materials & experimental systems

n/a	Involved in the study	
☐	☒	Antibodies
☐	☒	Eukaryotic cell lines
☒	☐	Palaeontology and archaeology
☐	☒	Animals and other organisms
☐	☒	Human research participants
☒	☐	Clinical data
☐	☒	Dual use research of concern

Methods

n/a	Involved in the study	
☒	☐	ChIP-seq
☐	☒	Flow cytometry
☒	☐	MRI-based neuroimaging

Antibodies

Antibodies used	Antibodies used for Flow Cytometry Staining: Anti-mouse H-2Kd (Mouse IgG2a, 1:100, BioLegend, 116607); Anti-human TEM8 antibody RB9075 (Rabbit IgG, 1:50, customized); Anti-FLAG (Rabbit IgG, 1:50, Sigma Aldrich, F7425).<br>Antibodies used for Western Blot: Anti-human TEM8 antibody RB9075 (Rabbit IgG, 1:500, customized); Anti-FLAG (Rabbit IgG, 1:1000, Sigma Aldrich, F7425); Anti-HA (Rabbit IgG, 1:1000, CST, 3724); ASB10 (Mouse IgG, 1:1000, Novus, H00136371-M02); Ubiquitin (Mouse IgG1, 1:500, Santa Cruz, sc-8017); SMAD5 (Rabbit IgG, 1:1000, CST, 12534); p-SMAD1/5 (Rabbit IgG, 1:1000, CST, 9516); SMAD1 (Rabbit IgG, 1:1000, CST, 6944); p-SMAD1 (Rabbit IgG, 1:1000, CST, 5753); p-SMAD2 (Rabbit IgG, 1:1000, CST, 3108); p-SMAD3 (Rabbit IgG, 1:1000, CST, 9520); SMAD2/3 (Rabbit IgG, 1:1000, CST, 8685); IκBα (Rabbit IgG, 1:1000, CST, 4812); p-IκBα (Rabbit IgG, 1:1000, CST, 2859); p65 (Rabbit IgG, 1:1000, CST, 8242); p-p65 (Rabbit IgG, 1:1000, CST, 3033); JNK (Rabbit IgG, 1:1000, CST, 9252); p-JNK (Rabbit IgG, 1:1000, CST, 9251); p-AKT (Rabbit IgG, 1:1000, CST, 4060); p38 (Rabbit IgG, 1:1000, CST, 9212); p-p38 (Rabbit IgG, 1:1000, CST, 4631); β-catenin (Rabbit IgG, 1:1000, CST, 8480); non-p β-catenin (Rabbit IgG, 1:1000, CST, 8814); VE-cadherin (Mouse IgG1, 1:1000, Santa Cruz, sc-9989); EphA2 (Mouse IgG2b, 1:1000, Santa Cruz, sc-398832); GAPDH (Mouse IgG, 1:1000, TransGen, HC301); RhoC (Rabbit IgG, 1:1000, CST, 3430).<br>Antibodies used for immunostaining: Anti-human TEM8 antibody RB9075 (Rabbit IgG, 1:100, customized); FLAG (Rabbit IgG, 1:100, Sigma Aldrich, F7425); HA (Rabbit IgG, 1:100, CST, 3724); ASB10 (Mouse IgG, 1:100, Novus, H00136371-M02); CD31 (Rabbit IgG, 1:50, Abcam, ab28364; Mouse IgG1, 1:50, Santa Cruz, sc-376764; Rat IgG2a, 1:50, Santa Cruz, sc-18916); ERα (Mouse IgG, 1:100, Abcam, ab241557; Rabbit IgG, Zsbio, ZA-0102); RhoC (Rabbit IgG, 1:100, CST, 3430); EphA2 (Mouse IgG2b, 1:50, Santa Cruz, sc-398832); NG2 (Mouse IgG1, 1:50, Santa Cruz, sc-53389).<br>Second antibodies in this study: PE-conjugated donkey anti-rabbit IgG (1:200, Jackson ImmunoResearch, 711-116-152); APC-conjugated donkey anti-rabbit IgG (1:200, Jackson ImmunoResearch, 711-136-152); HRP-conjugated goat anti-mouse IgG (1:5000, TransGen, HS201-01); HRP conjugated goat anti-rabbit IgG (1:5000, TransGen, HS101-01); Alexa Fluor 488 goat anti-mouse IgG (1:200, Invitrogen, A11001); Alexa Fluor 488 goat anti-rabbit IgG (1:200, Invitrogen, A11008); Alexa Fluor 546 goat anti-mouse IgG (1:200, Invitrogen, A11003); Alexa Fluor 546 goat anti-rabbit IgG (1:200, Invitrogen, A11035); Alexa Fluor 555 goat anti-rat IgG (1:200, Invitrogen, A21434); Alexa Fluor 647 donkey anti-mouse IgG (1:200, Jackson ImmunoResearch, 715-606-150).
Validation	Validation of the use of FLAG antibody for human in Western blot, immunostaining, flow cytometry staining and IP has been provided by the manufacturer's website.<br>Validation of the use of HA antibody for human in Western blot, immunostaining and IP has been provided by the manufacturer's website and relevant citations.<br>Validation of the use of RhoC antibody for human in Western blot and IP has been provided by the manufacturer's website.<br>Validation of the use of H-2Kd antibody for mouse in flow cytometry staining has been provided by the manufacturer's website.<br>Validation of the use of ASB10 antibody for human in Western blot has been provided by the manufacturer's website. The use of ASB10 antibody for human in immunostaining has been validated by staining of the ASB10 overexpression xenografts,.<br>Validation of the use of antibodies (including Ubiquitin, SMAD5, p-SMAD1/5, SMAD1, p-SMAD1, SMAD2/3, p-SMAD2, p-SMAD3, IκBα, p- IκBα, P65, p-P65, JNK, p-JNK, p-AKT, P38, p-P38, β-catenin, non-p β-catenin, VE-Cadherin and GAPDH) for human in Westen blot have been provided by the manufacturers' website.<br>Validation of the use of EphA2 antibody for human in Western blot and immunostaining has been provided by the manufacturer's website.<br>Validation of the use of CD31 antibodies for human and mouse in immunostaining have been provided by the manufacturer's website.<br>Validation of the use of ERα antibodies for human in immunostaining have been provided by the manufacturer's website.<br>Validation of the use of NG2 antibody for mouse in immunostaining have been provided by the manufacturer's website.

Eukaryotic cell lines

Policy information about cell lines

Cell line source(s)	Human breast cancer cell lines SUM149 and SUM159 were got from Asterland Bioscience; human breast cancer cell lines (MDA-MB-231, MDA-MB-231lung, and MCF-7) and 293T cell lines were purchased from ATCC (Manassas, Virginia),
Authentication	STR testing
Mycoplasma contamination	All cell lines were tested to be mycoplasma negative.
Commonly misidentified lines<br>(See ICLAC register)	All cell lines used in this study are not commonly misidentified cell lines.

Animals and other organisms

Policy information about studies involving animals; ARRIVE guidelines recommended for reporting animal research

Laboratory animals	Three to four-week-old female Nude mice and NOD/SCID mice were purchased from Charles river (Beijing, China) and housed in standard animal cages under a Specific-pathogen-free (SPF) facility at 23-25°C on a 12-h light/dark cycle in the Department of Laboratory Animal Science of Fudan University.
Wild animals	No wild animals was used in this study.
Field-collected samples	No field collected samples were used in the study.

Ethics oversight | Animal experiments were approved according to the experimental animal guidelines of the Care and Use of Laboratory Animals of Fudan University and approved by the Fudan University Shanghai Cancer Center Institutional Review Board (JS-082).

Note that full information on the approval of the study protocol must also be provided in the manuscript.

Human research participants

Policy information about studies involving human research participants

Population characteristics | *Describe the covariate-relevant population characteristics of the human research participants (e.g. age, gender, genotypic information, past and current diagnosis and treatment categories). If you filled out the behavioural & social sciences study design questions and have nothing to add here, write "See above."*

Recruitment | *Describe how participants were recruited. Outline any potential self-selection bias or other biases that may be present and how these are likely to impact results.*

Ethics oversight | *Identify the organization(s) that approved the study protocol.*

Note that full information on the approval of the study protocol must also be provided in the manuscript.

Dual use research of concern

Policy information about dual use research of concern

Hazards

Could the accidental, deliberate or reckless misuse of agents or technologies generated in the work, or the application of information presented in the manuscript, pose a threat to:

No	Yes	
☒	☐	Public health
☒	☐	National security
☒	☐	Crops and/or livestock
☒	☐	Ecosystems
☒	☐	Any other significant area

Experiments of concern

Does the work involve any of these experiments of concern:

No	Yes	
☒	☐	Demonstrate how to render a vaccine ineffective
☒	☐	Confer resistance to therapeutically useful antibiotics or antiviral agents
☒	☐	Enhance the virulence of a pathogen or render a nonpathogen virulent
☒	☐	Increase transmissibility of a pathogen
☒	☐	Alter the host range of a pathogen
☒	☐	Enable evasion of diagnostic/detection modalities
☒	☐	Enable the weaponization of a biological agent or toxin
☒	☐	Any other potentially harmful combination of experiments and agents

Flow Cytometry

Plots

Confirm that:

☒ The axis labels state the marker and fluorochrome used (e.g. CD4-FITC).

☒ The axis scales are clearly visible. Include numbers along axes only for bottom left plot of group (a 'group' is an analysis of identical markers).

☒ All plots are contour plots with outliers or pseudocolor plots.

☒ A numerical value for number of cells or percentage (with statistics) is provided.

Methodology

Sample preparation | Xenografted tumors or PDX harvested from mice were minced into small pieces and suspended in collagenase-hyaluronidase digestion solution (StemCell Technologies). The tumor pieces were put into shaker and digested at 37°C for 1 hour. Cell

	suspension was obtained after filtering with 40μm filter. The ammonium chloride solution (StemCell Technologies) was used for lysis of erythrocytes for 2 minutes at RT. The cell aggregates were obtained after centrifuging at 300 g for 5 minutes and resuspended for subsequent experiments.<br>For ALDEFLUOR assay (StemCell Technologies), dissociated single cells were suspended in ALDEFLUOR buffer containing ALDEFLUOR substrate BAAA and incubated at 37 °C for 40 minutes with or without DEAB. For single cells isolated from xenografted tumors, PE-conjugated anti-mouse H2kd antibody (Biolegend, 1:100) were used to discriminate human breast tumor cells from mouse cells. For TEM8 or FLAG-tag staining, single cells were stained with TEM8 antibody RB9075 or anti-FLAG antibody as the primary antibody (dilution at 1:50, the same isotype IgG used as the negative control) and APC- or PE-conjugated donkey anti-rabbit IgG (Jackson ImmunoResearch) as the secondary antibody (dilution at 1:200) on ice for 30 minutes.
Instrument	MoFlo Astrios instrument (Beckman Coulter, Brea, USA)
Software	Summit 6.3
Cell population abundance	Around 10^6 cells were prepared for each sample, and 10^4 cells were analyzed by Flow cytometer.
Gating strategy	The gating strategy (FSC-A vs SSC-A)was used to exclude cell debris and aggregates. The same gating strategy were applied to both control and experiment conditions.

☒ Tick this box to confirm that a figure exemplifying the gating strategy is provided in the Supplementary Information.
